# Serum YKL-40 in Non-ST-Segment Elevation Acute Coronary Syndrome: A Prospective Exploratory Emergency Department Study

**DOI:** 10.3390/medicina62081482

**Published:** 2026-08-01

**Authors:** Ece Zabun, Mustafa Burak Sayhan, Eray Çeliktürk, Satuk Buğra Han Bozatlı, Rıza Serttaş, Esin Seçgin Sayhan

**Affiliations:** 1 Department of Emergency Medicine, Medicine Faculty, Trakya University, Edirne 22030, Turkey; 2 Emergency Department, Edirne Sultan 1st Murat State Hospital, Edirne 22030, Turkey; 3 Department of Medical Biology, Medicine Faculty, Trakya University, Edirne 22030, Turkey; serttasriza@outlook.com; 4 Department of Public Health, Medicine Faculty, Trakya University, Edirne 22030, Turkey

**Keywords:** non-ST-segment elevation acute coronary syndrome, emergency department, YKL-40, biomarker, unstable angina, revascularization, inflammation

## Abstract

*Background and Objectives*: Non-ST-segment elevation acute coronary syndrome (NSTE-ACS) is a heterogeneous emergency condition in which early diagnostic assessment may be challenging. YKL-40 is associated with vascular inflammation and extracellular matrix remodeling, but its age-independent diagnostic relevance in NSTE-ACS remains uncertain. We evaluated the association and discriminatory performance of serum YKL-40 in patients with adjudicated NSTE-ACS versus non-ACS chest-pain controls; subtype discrimination, revascularization, and correlations with routinely measured inflammatory indices were explored as secondary outcomes. *Materials and Methods*: This prospective, single-center observational study included 88 adults presenting to the emergency department with chest pain between December 2024 and March 2025. Sixty had adjudicated NSTE-ACS, including 32 with non-ST-segment elevation myocardial infarction and 28 with unstable angina, while 28 constituted the non-ACS control group. Serum YKL-40 was measured at presentation using an enzyme-linked immunosorbent assay. Patient–control discrimination was assessed using receiver operating characteristic analysis, logistic regression, and bootstrap internal validation. Exploratory sensitivity analyses included Firth penalized regression and comparisons restricted to overlapping age ranges. *Results*: In the unadjusted analysis, serum YKL-40 concentrations were higher in the NSTE-ACS group than in non-ACS controls (median, 832.1 vs. 552.6 ng/L; *p* = 0.002). Log-transformed YKL-40 yielded an area under the curve of 0.709 (95% CI, 0.599–0.819; *p* < 0.001) and was associated with NSTE-ACS status (OR per 1-standard-deviation increase, 1.89; 95% CI, 1.15–3.12; *p* = 0.013). After adjustment for age, the association was attenuated and no longer statistically significant (OR, 1.58; 95% CI, 0.92–2.71; *p* = 0.100). Models additionally accounting for renal function and cardiovascular risk factors, together with analyses restricted to overlapping age ranges, yielded consistent findings. YKL-40 showed limited discrimination between NSTEMI and unstable angina (AUC, 0.527; 95% CI, 0.376–0.678), was not significantly associated with revascularization (OR, 1.76; 95% CI, 0.86–3.59; *p* = 0.120; AUC, 0.614), and was not significantly correlated with C-reactive protein, white blood cell count, or the neutrophil-to-lymphocyte ratio. *Conclusions*: The unadjusted association between YKL-40 and NSTE-ACS was attenuated after accounting for age and other baseline differences. These findings do not establish an age-independent diagnostic role or support the routine diagnostic use of YKL-40 in NSTE-ACS.

## 1. Introduction

Acute coronary syndrome is a heterogeneous clinical condition arising predominantly from atherosclerotic plaque disruption and thrombosis and requires prompt diagnostic assessment in the emergency department [1]. Non-ST-segment elevation acute coronary syndrome (NSTE-ACS), encompassing non-ST-segment elevation myocardial infarction (NSTEMI) and unstable angina, presents particular diagnostic and management challenges because clinical manifestations and early test findings may overlap [1,2]. Contemporary assessment relies primarily on clinical evaluation, serial electrocardiography, and high-sensitivity cardiac troponin measurements. Although cardiac troponin provides highly sensitive evidence of myocardial injury, it does not directly reflect the inflammatory and extracellular matrix-related processes involved in plaque instability [1,2,3,4].

Inflammation, macrophage and neutrophil activation, and extracellular matrix remodeling contribute substantially to atherosclerotic plaque progression and destabilization [3,4,5]. Chitinase-3-like protein 1, commonly known as YKL-40, is a glycoprotein released by macrophages, neutrophils, and vascular smooth-muscle cells and has been associated with inflammation, cellular proliferation, angiogenesis, and extracellular matrix remodeling. These biological properties suggest that circulating YKL-40 may reflect a pathophysiological component distinct from myocardial injury measured by cardiac troponin. Nevertheless, biological plausibility alone does not establish diagnostic usefulness, and circulating YKL-40 may also be influenced by age, renal function, and chronic disease burden [6].

Previous studies have linked elevated YKL-40 concentrations to ST-segment elevation myocardial infarction, adverse outcomes following acute coronary syndrome, and angiographically assessed coronary atherosclerotic burden [7,8,9,10]. However, its discriminatory performance in NSTE-ACS, its association after accounting for important baseline differences, and its relationships with clinical subtype and revascularization remain insufficiently characterized. In this prospective observational study, we evaluated the association and discriminatory performance of serum YKL-40 in patients with adjudicated NSTE-ACS compared with non-ACS chest-pain controls. Its ability to distinguish NSTEMI from unstable angina, its association with revascularization, and its correlations with routinely measured inflammatory indices were examined as exploratory secondary outcomes.

## 2. Materials and Methods

### 2.1. Study Design and Ethical Approval

This prospective, single-center observational study was conducted between December, 2024, and March, 2025, at the Adult Emergency Department of Trakya University Health Practice and Research Center. Ethical approval was obtained from the Scientific Research Ethics Committee of Trakya University Faculty of Medicine (Approval No: 20/21; 2 December 2024). Written informed consent was obtained from all participants, and the study was conducted in accordance with the Declaration of Helsinki.

### 2.2. Study Population and Group Definitions

During the study period, 1308 emergency department presentations with suspected NSTE-ACS were assessed for eligibility. Consecutive patients presenting with chest pain who were diagnosed with NSTE-ACS on the basis of guideline-concordant clinical assessment, serial electrocardiography, and serial high-sensitivity cardiac troponin testing were eligible for inclusion. Exclusion criteria were ST-segment elevation myocardial infarction, active infection, systemic or chronic inflammatory disease, malignancy, and failure to provide written informed consent.

For verification of the participant flow, a structured source-data audit was conducted using emergency department electronic medical records, cardiology consultation records, laboratory sampling records, study case-report forms, and written informed-consent documents. These records were used to reconstruct the screening and participant-selection process throughout the study period. The reconstructed screening dataset was independently reviewed by two investigators, and any discrepancies were resolved by re-examination of the relevant source records and consensus. The final screening counts were cross-checked against the study database and the definitive list of enrolled participants.

Unstable angina was defined as ischemic chest pain occurring at rest, of new onset, or showing a recent increase in frequency, duration, or severity, without a serial high-sensitivity cardiac troponin pattern consistent with acute myocardial infarction. Serial troponin results were interpreted according to the assay-specific 99th-percentile upper reference limit and the institutional 0–2-h delta criterion described in Section 2.4. When available, the diagnosis was supported by new or dynamic ST-segment depression or T-wave inversion, a new regional wall-motion abnormality, objective evidence of ischemia on noninvasive testing, and/or clinically significant coronary artery stenosis on coronary angiography. Coronary angiography was not mandatory for all patients with unstable angina and was performed when clinically indicated; however, detailed angiographic findings were not systematically captured in the study database.

Final NSTE-ACS classification, including the distinction between NSTEMI and unstable angina, was established by consensus between the attending emergency physician and a cardiologist after review of all available clinical, serial electrocardiographic, cardiac troponin, and imaging data. Both physicians were blinded to the serum YKL-40 results, and consensus was reached in all cases.

The NSTE-ACS group comprised 32 patients with NSTEMI and 28 with unstable angina. The non-ACS control group was prospectively recruited from eligible consenting patients who presented consecutively to the emergency department with chest pain during the study period and in whom acute coronary syndrome was excluded after completion of the emergency department diagnostic evaluation. Control recruitment continued until the prespecified target sample size of 28 participants was reached. No individual matching procedure was used between the NSTE-ACS and control groups.

Acute coronary syndrome was excluded through repeated clinical assessment, serial electrocardiography, and serial high-sensitivity cardiac troponin I measurements. Patients remained under clinical observation until the diagnostic evaluation was completed. Cardiology consultation and additional cardiac investigations were performed when clinically indicated. Final non-ACS diagnoses were not recorded in predefined diagnostic categories in the study database.

### 2.3. Data Collection

Demographic characteristics, cardiovascular risk factors and comorbidities—including hypertension, diabetes mellitus, smoking status, and hyperlipidemia—estimated glomerular filtration rate (eGFR), cardiac troponin I concentrations, electrocardiographic findings, and echocardiographic findings including left ventricular ejection fraction were extracted from the electronic medical records and patient files. Serum YKL-40 was measured specifically for this study. C-reactive protein, white blood cell, neutrophil, and lymphocyte counts were obtained from routine laboratory records, and the neutrophil-to-lymphocyte ratio was calculated. Revascularization was defined as percutaneous coronary intervention or coronary artery bypass grafting performed during the index hospitalization and was verified from hospital records. All study data were de-identified before statistical analysis.

### 2.4. Laboratory Methods

High-sensitivity cardiac troponin I (hs-cTnI) was measured as part of the routine clinical evaluation using the ADVIA Centaur High-Sensitivity Troponin I assay on the ADVIA Centaur CP Immunoassay System (Siemens Healthineers, Erlangen, Germany). The sex-specific 99th-percentile upper reference limits were 36.99 ng/L for women and 57.27 ng/L for men. Measurements were obtained at presentation and 2 h later. Under the institutional assay-specific protocol in use during the study period, an absolute change of ≥20 ng/L between the 0- and 2-h measurements was considered significant. Troponin results were interpreted in conjunction with the clinical presentation and serial electrocardiographic findings [1].

For study-specific YKL-40 measurement, venous blood was obtained from all participants at emergency department presentation before treatment was initiated. Samples were collected in serum separator tubes, allowed to clot at room temperature, and centrifuged at 3000 rpm for 10 min. The separated serum was aliquoted and stored at −80 °C until analysis. Serum YKL-40 concentrations were determined using a commercially available enzyme-linked immunosorbent assay kit (ELISA; A.B.T., Cat. No. ABT1021Hu, Istanbul, Türkiye) in accordance with the manufacturer’s instructions. Optical density was measured at 450 nm using a microplate reader (Thermo Fisher Scientific, Vantaa, Finland). Stored serum samples were assayed in duplicate within the same analytical batch, and the mean of the duplicate measurements was used for statistical analysis. Laboratory personnel performing the YKL-40 assays were blinded to participants’ clinical information and group allocation. The manufacturer-reported intra-assay and inter-assay coefficients of variation were each < 10%.

### 2.5. Outcome Measures

The primary outcome was the discriminatory performance of serum YKL-40 for distinguishing patients with adjudicated NSTE-ACS from non-ACS chest-pain controls. Secondary outcomes included the ability of YKL-40 to differentiate NSTEMI from unstable angina, its association with revascularization, and its correlations with C-reactive protein, white blood cell count, and the neutrophil-to-lymphocyte ratio. All secondary analyses were considered exploratory.

### 2.6. Statistical Analysis

Statistical analyses were performed using IBM SPSS Statistics, version 26.0 (IBM Corp., Armonk, NY, USA), and MedCalc Statistical Software, version 20.0 (MedCalc Software Ltd., Ostend, Belgium). The required sample size was estimated before data collection using G*Power(version 3.1). Based on the patient–control difference in serum YKL-40 reported by Fang et al. [7], a standardized effect size of 0.62 was assumed. With a type I error rate of 0.05 and statistical power of 80%, the minimum required total sample size was calculated as 84 participants. This calculation was based on the anticipated between-group difference in YKL-40 rather than the area under the receiver operating characteristic curve. No separate power calculations were performed for the NSTEMI–unstable angina or revascularization analyses; these secondary analyses were therefore considered exploratory.

The distribution of continuous variables was assessed using the Shapiro–Wilk test. Normally distributed variables are summarized as mean ± standard deviation, whereas non-normally distributed variables are presented as median and interquartile range. Two-group comparisons were performed using the independent-samples Student’s *t*-test or Mann–Whitney U test, as appropriate. Comparisons across three groups were conducted using one-way analysis of variance or the Kruskal–Wallis test. Categorical variables were compared using the chi-square test or Fisher’s exact test. Associations between YKL-40 and inflammatory parameters were assessed using Spearman’s rank correlation coefficients. Because YKL-40 showed a right-skewed distribution, values were natural-log-transformed before ROC and regression analyses. Discriminatory performance was evaluated using ROC curve analysis, and AUCs were reported with 95% confidence intervals estimated using the DeLong method.

Logistic regression models were used to estimate odds ratios with 95% confidence intervals per 1-standard-deviation increase in log-transformed YKL-40. Given the limited sample size, the primary multivariable model was restricted to age and log-transformed YKL-40 to reduce the risk of overfitting. Age was included because it is an established determinant of circulating YKL-40 and showed a marked imbalance between the study groups; accordingly, this model was interpreted as age-adjusted rather than fully adjusted. In exploratory sensitivity analyses, Firth penalized logistic regression was used to reduce small-sample bias. Progressively adjusted models included age and eGFR, followed by age, eGFR, hypertension, and diabetes mellitus, with penalized odds ratios and profile-likelihood 95% confidence intervals reported. Additional sensitivity analyses were conducted within overlapping age ranges common to the patient and control groups. Model optimism was assessed using 1000 bootstrap resamples, and optimism-corrected AUCs were calculated. All tests were two-sided, and *p* < 0.05 was considered statistically significant. No adjustment was made for multiple comparisons; *p* values from secondary and sensitivity analyses were interpreted as nominal. The study was reported in accordance with the Strengthening the Reporting of Observational Studies in Epidemiology guidelines.

## 3. Results

### 3.1. Study Population and Clinical Characteristics

**Figure 1:**
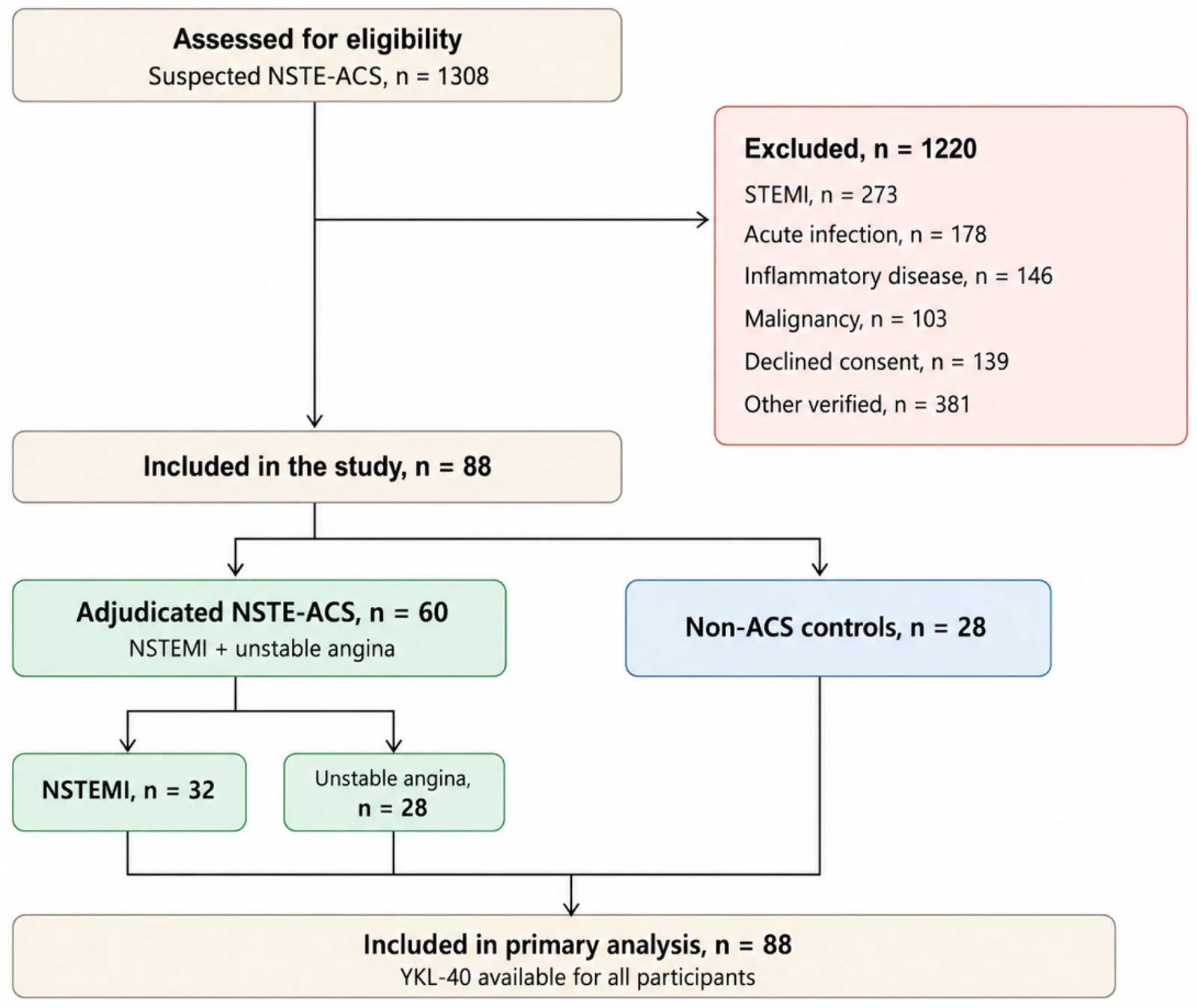
Study flow diagram of participant screening, exclusion, adjudication, and inclusion in the primary analysis.

A total of 88 participants were included in the study. Of these, 60 had adjudicated NSTE-ACS, including 32 patients with NSTEMI and 28 with unstable angina, while 28 participants constituted the non-ACS control group. The groups differed significantly in age (*p* < 0.001), whereas the sex distribution was similar across groups (*p* = 0.720). Baseline demographic and clinical characteristics are summarized in Table 1. The study flow is shown in Figure 1.

### 3.2. Comparison of YKL-40 Levels Between Groups

In the unadjusted analysis, serum YKL-40 concentrations were higher in the NSTE-ACS group than in the non-ACS control group (median, 832.1 vs. 552.6 ng/L; *p* = 0.002).

### 3.3. Discrimination Between NSTE-ACS and Non-ACS Controls

**Figure 2:**
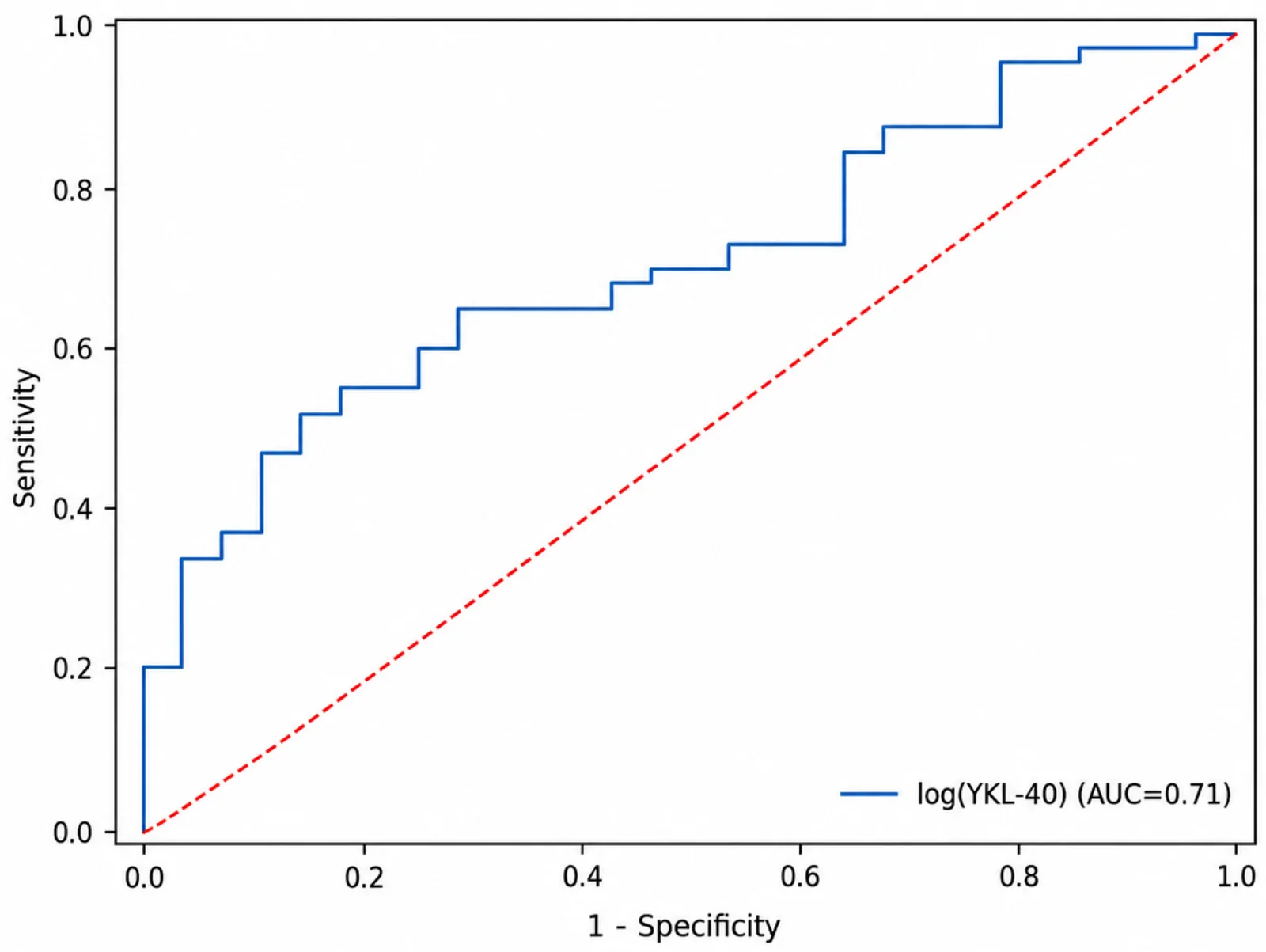
Receiver operating characteristic curve for discrimination between patients with NSTE-ACS and non-ACS controls using log-transformed YKL-40. The 95% confidence interval for the AUC was estimated using the DeLong method. The red dashed cross represents non-discriminatory performance, corresponding to an AUC of 0.50.

Log-transformed YKL-40 showed moderate discrimination between patients with NSTE-ACS and non-ACS controls, with an AUC of 0.709 (95% CI, 0.599–0.819; *p* < 0.001). The corresponding ROC curve is shown in Figure 2. In unadjusted logistic regression, each 1-standard-deviation increase in log-transformed YKL-40 was associated with higher odds of NSTE-ACS status (OR, 1.89; 95% CI, 1.15–3.12; *p* = 0.013). Internal validation using 1000 bootstrap resamples yielded an optimism-corrected AUC of 0.708. After adjustment for age, the association between log-transformed YKL-40 and NSTE-ACS status was attenuated and no longer statistically significant (OR, 1.58; 95% CI, 0.92–2.71; *p* = 0.100), whereas age remained independently associated with NSTE-ACS status (OR per year, 1.10; 95% CI, 1.05–1.15; *p* < 0.001). The crude and age-adjusted model results are summarized in Table 2.

### 3.4. Sensitivity Analyses

In exploratory analyses addressing potential confounding beyond age, the association between log-transformed YKL-40 and NSTE-ACS status weakened with progressive covariate adjustment. In Firth penalized logistic regression, the OR per 1-standard-deviation increase in log-transformed YKL-40 was 1.53 (95% CI, 0.92–2.62; *p* = 0.098) after adjustment for age, 1.42 (95% CI, 0.87–2.40; *p* = 0.150) after additional adjustment for eGFR, and 1.17 (95% CI, 0.67–2.02; *p* = 0.570) after further inclusion of hypertension and diabetes mellitus. A conventional maximum-likelihood model adjusted for age and eGFR yielded a comparable estimate (OR, 1.47; 95% CI, 0.87–2.49; *p* = 0.150).

When the analysis was restricted to the overlapping age range of 31–67 years (39 patients with NSTE-ACS and 19 non-ACS controls), log-transformed YKL-40 showed limited discriminatory performance (AUC, 0.62), and its unadjusted association with NSTE-ACS status was not statistically significant (OR per 1-standard-deviation increase, 1.39; 95% CI, 0.81–2.39; *p* = 0.230). Findings were similar in the narrower 45–65-year subgroup (*n* = 38; OR, 1.38; 95% CI, 0.69–2.79; *p* = 0.360). Detailed results are presented in Table 3.

### 3.5. Clinical Subtype Discrimination: NSTEMI Versus Unstable Angina

**Figure 3:**
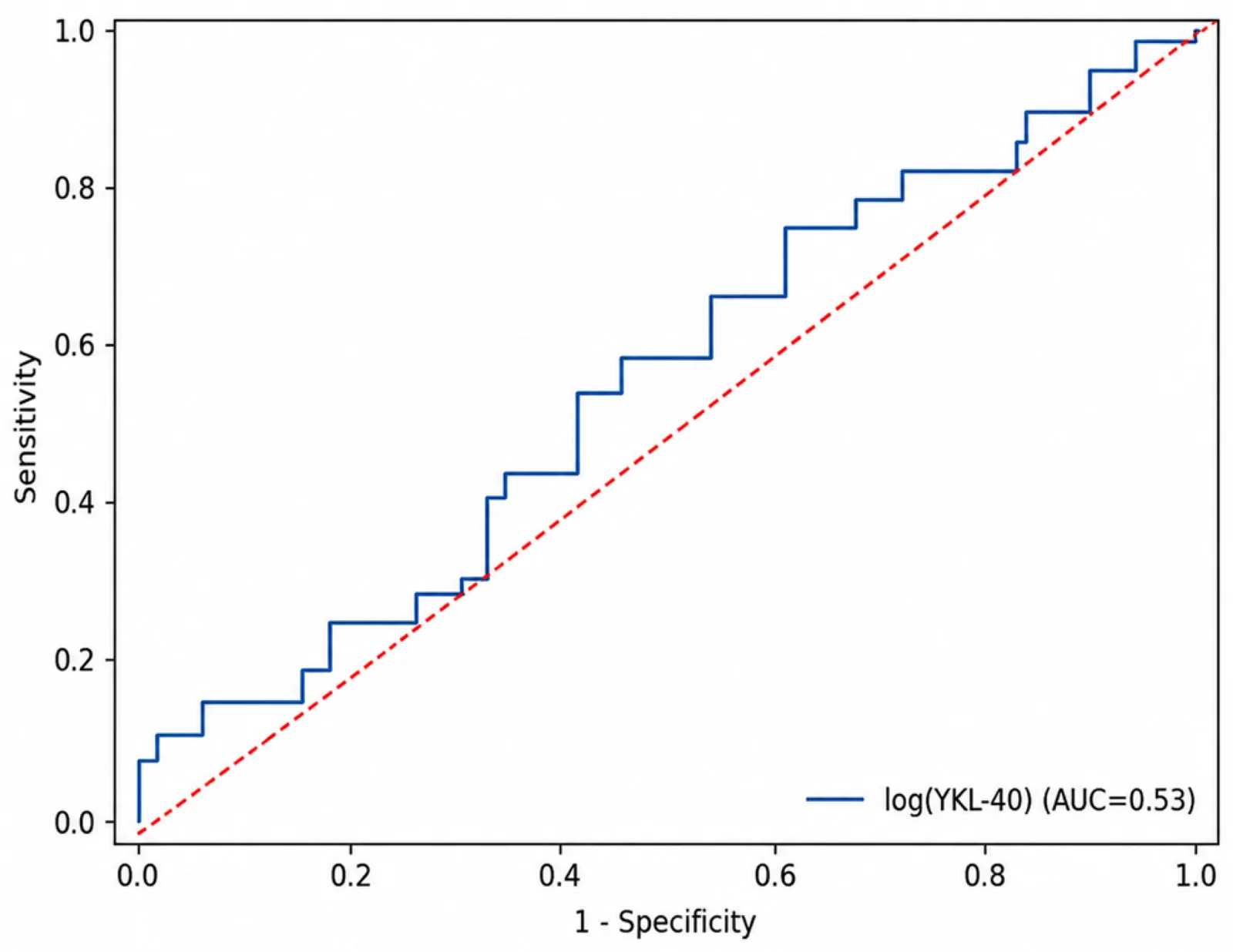
Receiver operating characteristic curve for discrimination between NSTEMI and unstable angina using log-transformed YKL-40. The 95% confidence interval for the AUC was estimated using the DeLong method. The red dashed cross represents non-discriminatory performance, corresponding to an AUC of 0.50.

Median serum YKL-40 concentrations were 875.43 ng/L (IQR, 486.14–1001.14) in patients with NSTEMI and 768.29 ng/L (IQR, 402.57–1064.36) in those with unstable angina, with no significant difference between the subgroups (*p* = 0.728). In this exploratory analysis, log-transformed YKL-40 showed limited discrimination between NSTEMI and unstable angina (AUC, 0.527; 95% CI, 0.376–0.678). The corresponding ROC curve is shown in Figure 3.

### 3.6. Associations Between YKL-40 and Inflammatory Markers

Serum YKL-40 was not significantly correlated with C-reactive protein (Spearman’s ρ = 0.044; *p* = 0.681), white blood cell count (ρ = −0.016; *p* = 0.882), or the neutrophil-to-lymphocyte ratio (ρ = 0.122; *p* = 0.258).

### 3.7. Association Between YKL-40 and Revascularization

Among the 60 patients with NSTE-ACS, 25 (41.7%) underwent revascularization during the index hospitalization. Log-transformed YKL-40 was not significantly associated with revascularization (OR per 1-standard-deviation increase, 1.76; 95% CI, 0.86–3.59; *p* = 0.120). Its discriminatory performance was limited (AUC, 0.614; 95% CI, 0.469–0.759), with an optimism-corrected AUC of 0.608. The results are summarized in Table 4.

## 4. Discussion

The principal finding of this prospective study was that serum YKL-40 concentrations were higher in patients with NSTE-ACS than in non-ACS controls in the unadjusted analysis, but the association was attenuated after adjustment for age and remained non-significant in exploratory analyses accounting for renal function and cardiovascular risk factors or restricting the comparison to overlapping age ranges. YKL-40 also showed limited discrimination between NSTEMI and unstable angina, was not significantly associated with revascularization, and was not significantly correlated with C-reactive protein, white blood cell count, or the neutrophil-to-lymphocyte ratio. Overall, these findings do not demonstrate an age-independent diagnostic role for YKL-40 in this cohort.

The primary patient–control comparison should be interpreted in light of the substantial baseline differences between the groups. Compared with the NSTE-ACS group, the non-ACS controls were younger, had fewer cardiovascular risk factors, and had better renal function. Circulating YKL-40 concentrations increase with age and may also be influenced by chronic disease burden and renal function [6]. Although age was included in the primary multivariable model, residual confounding could not be fully excluded. The further attenuation of the association in Firth penalized models and the absence of a significant association in the overlapping-age analyses suggest that the crude difference was influenced, at least partly, by baseline imbalance rather than by an NSTE-ACS-specific biomarker signal. Moreover, because the study compared patients with adjudicated NSTE-ACS with non-ACS controls rather than evaluating YKL-40 within an undifferentiated chest-pain pathway, the observed AUC should not be interpreted as evidence of real-time rule-in or rule-out performance.

Inflammation, plaque healing, extracellular matrix remodeling, and trained immune responses contribute to the development and progression of atherosclerotic plaque instability [3,4,5,11,12]. YKL-40 is released by macrophages, neutrophils, and vascular smooth-muscle cells and has been associated with inflammatory activity, angiogenesis, and extracellular matrix remodeling [6]. Previous studies have linked circulating YKL-40 to STEMI, mortality after acute coronary syndrome, and angiographically assessed coronary atherosclerotic burden [7,8,9,10]. In the present cohort, however, YKL-40 was not significantly correlated with routinely measured systemic inflammatory indices. This finding suggests that circulating YKL-40 may not simply parallel C-reactive protein, white blood cell count, or the neutrophil-to-lymphocyte ratio; nevertheless, the absence of correlation should not be interpreted as excluding a potential biological role. Differences in disease severity, study endpoints, case mix, and biomarker sampling strategies may also help explain the divergence from previous reports, which primarily addressed long-term outcomes or anatomical disease burden rather than early diagnostic discrimination in the emergency department.

The secondary findings also warrant cautious interpretation. The limited discrimination between NSTEMI and unstable angina does not support the use of a single admission YKL-40 measurement for clinical subtype classification. Similarly, YKL-40 was not significantly associated with revascularization, an outcome influenced by coronary anatomy; ischemic burden; clinical presentation; comorbidities; and treatment decisions [1,13]. Given the limited subgroup sizes, these analyses should be regarded as exploratory. High-sensitivity cardiac troponin-based rule-in and rule-out algorithms remain the established diagnostic approach in emergency department practice [13], and the present results do not support routine YKL-40 measurement for diagnostic or risk-stratification purposes. Future studies should enroll clinically comparable patients presenting with undifferentiated chest pain, standardize sampling relative to symptom onset, and evaluate serial YKL-40 measurements alongside established diagnostic pathways.

### Limitations

This study has several limitations. First, its single-center design may limit the generalizability of the findings. In addition, the marked differences in age, cardiovascular risk burden, and renal function between the NSTE-ACS and control groups complicate interpretation of the patient–control comparison. Residual confounding therefore cannot be fully excluded despite the age-adjusted and exploratory sensitivity analyses. The final non-ACS diagnoses in the control group were also not systematically recorded in predefined categories, limiting detailed characterization of the clinical heterogeneity of the control population.

Second, although the sample size was planned on the basis of the expected patient–control difference in YKL-40 concentrations, separate power calculations were not performed for the NSTEMI–unstable angina and revascularization analyses. These secondary analyses consequently had limited statistical precision. YKL-40 was measured only once at admission, and the interval between chest-pain onset and blood sampling was not systematically recorded; therefore, temporal biomarker changes and the potential influence of sampling-time variability could not be assessed. Long-term outcomes were not evaluated, precluding conclusions regarding prognostic value beyond the index hospitalization.

Finally, medication use at the time of sampling—including statins, corticosteroids, ACE inhibitors or angiotensin receptor blockers, and immunosuppressive agents—and family history of coronary artery disease were not systematically recorded, representing potential sources of residual confounding and indicating that the clinical risk profile was not fully characterized. Inflammatory assessment was limited to selected circulating markers, and imaging correlates of plaque phenotype were not evaluated. Detailed angiographic findings in patients with unstable angina were also not systematically captured, limiting assessment of the anatomical evidence supporting this classification.

## 5. Conclusions

In this prospective exploratory emergency department cohort, serum YKL-40 concentrations were higher in patients with NSTE-ACS than in non-ACS controls in the unadjusted analysis; however, the association was attenuated after adjustment for age and remained non-significant in exploratory sensitivity analyses accounting for renal function and cardiovascular risk factors. YKL-40 showed limited discrimination between NSTEMI and unstable angina and was not significantly associated with revascularization. These findings do not support an age-independent diagnostic role or routine use of YKL-40 for diagnostic or risk-stratification purposes in NSTE-ACS.

## Figures and Tables

**Table 1:** Baseline Characteristics Across Study Groups.

Variable	NSTEMI (*n* = 32)	UA (*n* = 28)	Non-ACS Control (*n* = 28)	*p* Value ^†^
Age, years	67.38 ± 14.05	56.43 ± 11.15	40.00 ± 16.16	<0.001
Male sex, *n* (%)	20 (62.5)	19 (67.9)	16 (57.1)	0.720
Hypertension, *n* (%)	24 (75.0)	7 (25.0)	2 (7.1)	<0.001
Diabetes mellitus, *n* (%)	14 (43.8)	5 (17.9)	4 (14.3)	0.017
Current smoking, *n* (%)	3 (9.4)	5 (17.9)	3 (10.7)	0.576
Hyperlipidemia, *n* (%)	4 (12.5)	3 (10.7)	2 (7.1)	0.788
eGFR, mL/min/1.73 m^2^	59.6 ± 31.5	81.3 ± 23.0	95.1 ± 14.0	<0.001
Admission hs-cTnI, ng/L	221.5 (96.0–3310.0)	4.95 (3.47–9.25)	3.30 (2.40–4.53)	<0.001

Footnotes: Values are presented as mean ± standard deviation, median (interquartile range), or number (percentage), as appropriate. ^†^ Overall *p* values represent comparisons among the NSTEMI, unstable angina, and non-ACS control groups and were calculated using one-way analysis of variance or the Kruskal–Wallis test for continuous variables and the chi-square test or Fisher’s exact test for categorical variables, as appropriate. NSTEMI, non-ST-segment elevation myocardial infarction; UA, unstable angina; eGFR, estimated glomerular filtration rate; hs-cTnI, high-sensitivity cardiac troponin I.

**Table 2:** Patient–Control Discrimination and Association of Log-Transformed YKL-40 in Crude and Age-Adjusted Models.

Model	AUC (95% CI)	Odds Ratio (95% CI) *	*p* Value	Optimism-Corrected AUC ^†^
Log(YKL-40)	0.709 (0.599–0.819)	1.89 (1.15–3.12)	0.013	0.708
Log(YKL-40) + Age ^‡^	0.862 (0.783–0.941)	1.58 (0.92–2.71)	0.100	0.851

Footnotes: AUC calculated using ROC analysis with the DeLong method. * Odds ratio derived from logistic regression per 1 standard deviation increase in log-transformed YKL-40. ^†^ Optimism-corrected AUC obtained using 1000 bootstrap resamples. ^‡^ For the age-adjusted model, the reported AUC represents the full model including age and log-transformed YKL-40; the odds ratio and *p* value refer to log-transformed YKL-40 after adjustment for age.

**Table 3:** Sensitivity Analyses of the Association Between Log-Transformed YKL-40 and NSTE-ACS Status.

Analysis	Model Specification or Age Restriction	*n*	OR per 1-SD Increase in Log-Transformed YKL-40 (95% CI)	*p* Value	AUC
Firth penalized logistic regression	Unadjusted	88	1.81 (1.16–3.08)	0.008	—
Firth penalized logistic regression	Adjusted for age	88	1.53 (0.92–2.62)	0.098	—
Firth penalized logistic regression	Adjusted for age and eGFR	88	1.42 (0.87–2.40)	0.150	—
Firth penalized logistic regression	Adjusted for age, eGFR, hypertension, and diabetes mellitus	87	1.17 (0.67–2.02)	0.570	—
Conventional maximum-likelihood logistic regression	Adjusted for age and eGFR	88	1.47 (0.87–2.49)	0.150	—
Overlapping-age analysis	Restricted to 31–67 years	58	1.39 (0.81–2.39)	0.230	0.620
Overlapping-age analysis	Restricted to 45–65 years	38	1.38 (0.69–2.79)	0.360	—

Footnotes: Odds ratios are reported per 1-standard-deviation increase in natural-log-transformed YKL-40. Firth penalized logistic regression was used to reduce small-sample bias, and profile-likelihood 95% confidence intervals are reported for the penalized models. The conventional maximum-likelihood model was included as a consistency analysis. Analyses restricted to overlapping age ranges were unadjusted and exploratory. The fully adjusted Firth model included 87 participants because of missing covariate data. Reported *p* values are two-sided and nominal, without adjustment for multiple comparisons. OR, odds ratio; CI, confidence interval; SD, standard deviation; eGFR, estimated glomerular filtration rate; NSTE-ACS, non-ST-segment elevation acute coronary syndrome; AUC, area under the receiver operating characteristic curve.

**Table 4:** Association and Discriminatory Performance of Log-Transformed YKL-40 for Revascularization.

Measure	Estimate (95% CI)	*p* Value	Optimism-Corrected AUC
Association with revascularization, OR per 1-SD increase	1.76 (0.86–3.59)	0.120	—
Discrimination for revascularization, AUC	0.614 (0.469–0.759)	—	0.608

Footnote: The OR is reported per 1-standard-deviation increase in natural-log-transformed YKL-40. The 95% confidence interval for the AUC was estimated using the DeLong method. The optimism-corrected AUC was obtained from 1000 bootstrap resamples. OR, odds ratio; AUC, area under the receiver operating characteristic curve; CI, confidence interval; SD, standard deviation.

## Data Availability

The data supporting the findings of this study are available from the corresponding author upon reasonable request. The data are not publicly available due to privacy and ethical restrictions related to patient information.
